# NcRNAs-mediated P2RX1 expression correlates with clinical outcomes and immune infiltration in patients with breast invasive carcinoma

**DOI:** 10.18632/aging.204087

**Published:** 2022-05-18

**Authors:** Yiyue Xu, Bing Zou, Bingjie Fan, Butuo Li, Jinming Yu, Linlin Wang, Jin Zhang

**Affiliations:** 1 3rd Department of Breast Cancer, China Tianjin Breast Cancer Prevention, Treatment and Research Center, Tianjin Medical University Cancer Institute and Hospital, Tianjin, P.R. China; 2 Key Laboratory of Breast Cancer Prevention and Therapy of Ministry of Education, Tianjin, P.R. China; 3 Key Laboratory of Cancer Prevention and Therapy, Tianjin, P.R. China; 4 Tianjin’s Clinical Research Center for Cancer, Tianjin, P.R. China; 5 Tianjin Medical University Cancer Institute and Hospital, National Clinical Research Center for Cancer, Tianjin, P.R. China; 6 Department of Radiation Oncology, Shandong Cancer Hospital and Institute, Shandong First Medical University and Shandong Academy of Medical Sciences, Jinan, P.R. China

**Keywords:** P2RX1, breast cancer, noncoding RNA, immune infiltration, prognosis

## Abstract

The development of novel treatments for breast invasive carcinoma (BC) has been stagnant. P2RX1, a member of the purinergic receptor family, has been found to have a prognostic impact in several tumors. Therefore, we analyzed the expression pattern of P2RX1 in pan-cancers including BC and its impact on survival and found that the expression level of P2RX1 was lower in BC compared with para-cancerous tissues, and higher P2RX1 expression indicated better prognoses. But real-time quantitative reverse transcription PCR (RT-qPCR) and Western blot detected that the P2RX1 expression in normal mammary epithelial cells was lower than that in tumor cells. Then we comprehensively analyzed the regulatory mechanism and protein-protein interaction network, and found that P2RX1 was significantly positively linked with immune cell infiltration and immune checkpoints.

## INTRODUCTION

Breast cancer is the most common cancer globally, and the leading cause of tumor-related deaths among women worldwide [1]. Despite the fact that great progress has been made in the development of therapies and predictive markers, like hormone receptor status, human epidermal growth factor receptor 2(Her-2) expression, and breast invasive carcinoma (BC) mutation status, there is insufficient knowledge on the understanding of the complicated pathophysiology of BC. Especially considering the coming era of immunotherapy, immune checkpoint inhibitors are shifting the paradigm of BC treatment [2, 3]. Nevertheless, only a few BC patients have benefited from immunotherapy [4]. PD-L1, CTLA-4, tumor microenvironment (TME), and tumor-infiltrating lymphocytes (TILs) status is usually considered biomarkers for immunotherapy. But the imperfect effect makes it an urgent need for novel immune-related prognostic biomarkers to guide clinicians in treating BC patients.

Adenosine triphosphate (ATP) is a basic element and important signal molecular of all living organisms, mainly generated via glycolysis and oxidative phosphorylation. Under normal physiological conditions, extracellular ATP exhibits a low concentration [5]. But in pathological situations such as inflammation or cancer, massive amounts of ATP are released into the extracellular environment, and the ATP concentration can be as high as several hundred micromoles [6]. P2RX1 belongs to a family of ATP-gated trimeric ion channels and is expressed on the surface of a variety of cells, including neutrophils, tumor cells, and smooth muscle cells [7–9]. When activated by extracellular ATP, P2RX1 can induce rapid Ca 2+, Na+, K+ influx across the cytoplasmic membrane, and lead to cell activation [10]. The expression of P2RX1 is abundant on TILs in some tumor types, so P2RX1 may be correlated with the TME [11]. Wang et al. found that activated P2X1R enhances neutrophil chemotaxis through the Rho-kinase signaling pathway (random cell migration) [10]. Neutrophil migration is inhibited in the P2X1-deficient mice [12]. Moreover, activating P2RX1 expressed by tumor cells also impacts tumor growth [13]. A previous study showed that the accumulation of immunosuppressive P2RX1-negative neutrophil subsets causes metastatic pancreatic cancer to evade antitumor immunity [14]. In lung cancer research, low P2RX1 expression was found to be associated with poor prognosis [15]. However, there remains a lack of comprehensive study on the expression, function, and mechanism of P2RX1 in BC, and its relationship with BC immune infiltration and clinical outcomes are still undetermined.

In this study, we first characterized the P2RX1 expression and association with outcomes by performing expression and survival analysis of pan-cancer samples from the database. Then we validated that P2RX1 was differentially expressed in two human breast cancer cell lines compared to normal human mammary epithelial cell line. In addition, we explored the non-coding RNAs (ncRNAs)-associated regulation of P2RX1 in BC, including microRNAs (miRNAs) and long non-coding RNAs (lncRNAs). Finally, we evaluated the relationship between P2RX1 and multiple TILs and immune checkpoints in BC. In summary, our study elucidates the important role of P2RX1 expression in BC and provides information regarding potential mechanisms for the interaction of P2RX1 with TME.

## RESULTS

### P2RX1 is down-regulated in a variety of tumors

**Figure 1:**
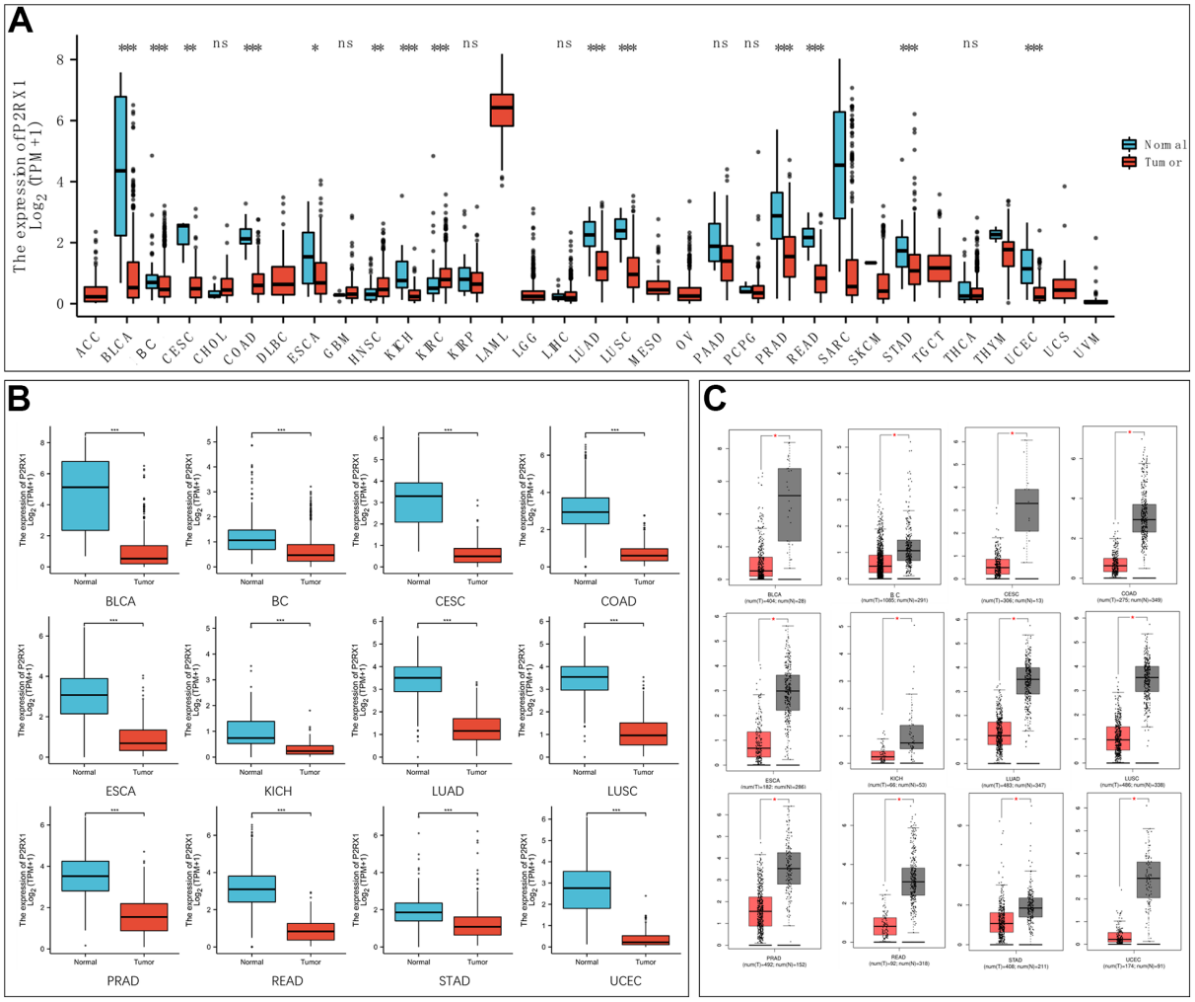
**Expression analysis of P2RX1 in pan-cancer.** (**A**) P2RX1 expression in 33 types of tumor tissues and paired adjacent normal tissues. (**B**) Differential expression analysis of P2RX1 based on TCGA and GTEx database. (**C**) P2RX1 expression in GEPIA database.

The mRNA expression of P2RX1 was analyzed to identify the differential expression in normal and tumor tissues. We first used the Cancer Genome Atlas (TCGA) database and found the mRNA levels of P2RX1 are comparatively lower in tumor tissues than paired adjacent normal tissues in bladder urothelial carcinoma (BLCA), BC, cervical squamous cell carcinoma and endocervical adenocarcinoma (CESC), colon adenocarcinoma (COAD), esophageal carcinoma (ESCA), kidney chromophobe (KICH), lung adenocarcinoma (LUAD), lung squamous cell carcinoma (LUSC), prostate adenocarcinoma (PRAD), rectum adenocarcinoma (READ), stomach adenocarcinoma (STAD), and uterine corpus endometrial carcinoma (UCEC), while higher in head and neck squamous cell carcinoma (HNSC) and kidney renal clear cell carcinoma (KIRC) (Figure 1A). Then we analyzed the P2RX1 expression between normal and tumor tissues using the TCGA database and the Genotype-Tissue Expression (GTEx) database, and similar results showed that P2RX1 is lowly expressed in a variety of tumors (Figure 1B). The Gene Expression Profiling Interactive Analysis (GEPIA) website further confirmed the differential expression (Figure 1C). This indicates that P2RX1 may be associated with an underlying carcinoma.

**Figure 2:**
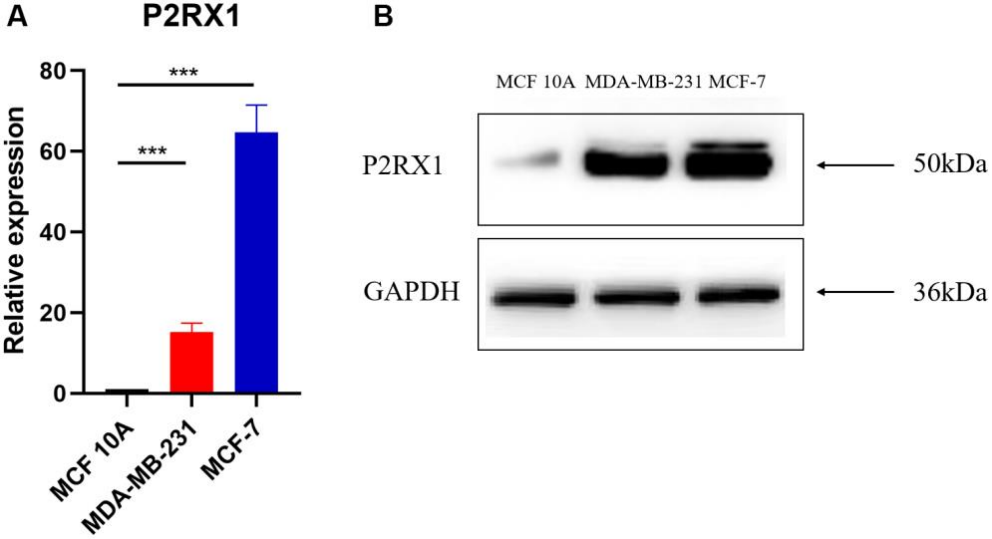
**P2RX1 expression in normal human mammary epithelial cell line and two human breast cancer cell lines.** (**A**) Analysis of P2RX1 expression by RT-qPCR. (**B**) Analysis of P2RX1 expression by Western blot.

The para-cancerous tissues are complex tissues composed of multiple cell types, including normal mammary epithelial cells, immune cells, and fibroblasts. To evaluate whether P2RX1 is highly expressed in mammary epithelial cells, we examined the matched breast tumor/normal pairs by RT-qPCR and Western blot. RT-qPCR demonstrated that the expression of P2RX1 in normal human mammary epithelial cells (MCF 10A) was significantly downregulated, compared with that in human breast cancer cells (MDA-MB-231, MCF-7) (Figure 2A). The Western blot result showed the same trend (Figure 2B).

### P2RX1 is significantly associated with prognosis in BC

**Figure 3:**
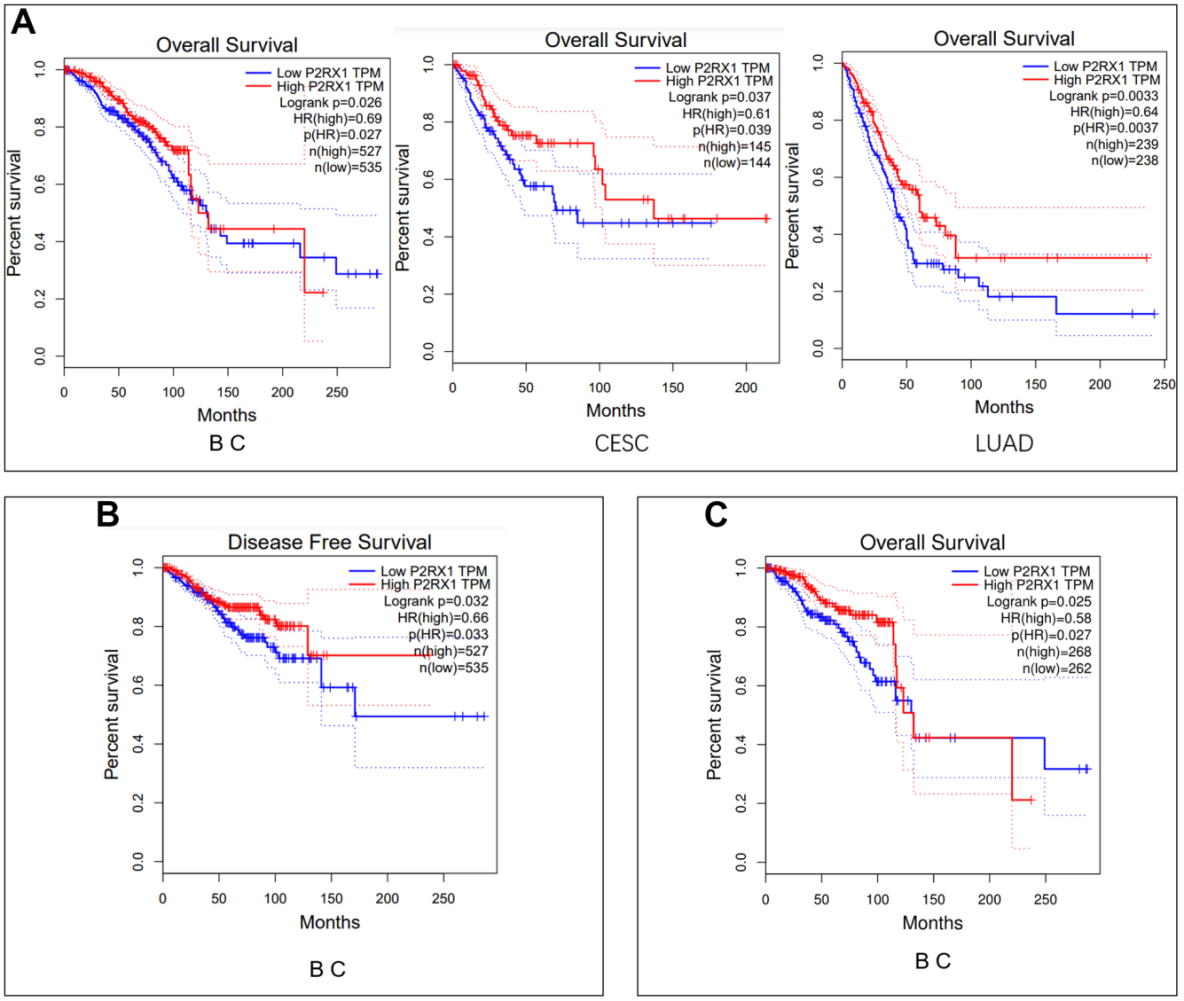
**Survival analysis.** (**A**) Overall survival analysis of P2RX1 expression in BC, CESC, and LUAD using the GIPIA platform. (**B**) Disease-free survival analysis of P2RX1 expression in BC using the GIPIA platform. (**C**) Overall survival analysis of P2RX1 expression in BC with the cut-off of the 25th and 75th percentile expression levels.

We evaluated the impact of P2RX1 on cancer prognosis among these 12 human cancers with differential expression of P2RX1 mRNA. Analysis of the GEPIA website manifested that high P2RX1 expression was correlated with better overall survival (OS) in BC, CESC and LUAD patients (p = 0.026, 0.035, and 0.0037, respectively) (Figure 3A). Besides, P2RX1 expression was also strongly associated with disease-free survival (DFS) in BC patients (p = 0.032) (Figure 3B). According to the survival analysis results, BC patients with low P2RX1 expression had 1.45 times the risk of OS and 1.52 times of risk of DFS than those with high P2RX1 expression. Further setting the cut-off to the 25th and 75th percentile expression levels of P2RX1 still showed that the high expression of P2RX1 was associated with better prognoses in BC patients (p = 0.025) (Figure 3C). Therefore, a low expression level of P2RX1 may be an unfavorable prognostic biomarker for BC patients.

### Prediction of upstream miRNA of P2RX1

**Figure 4:**
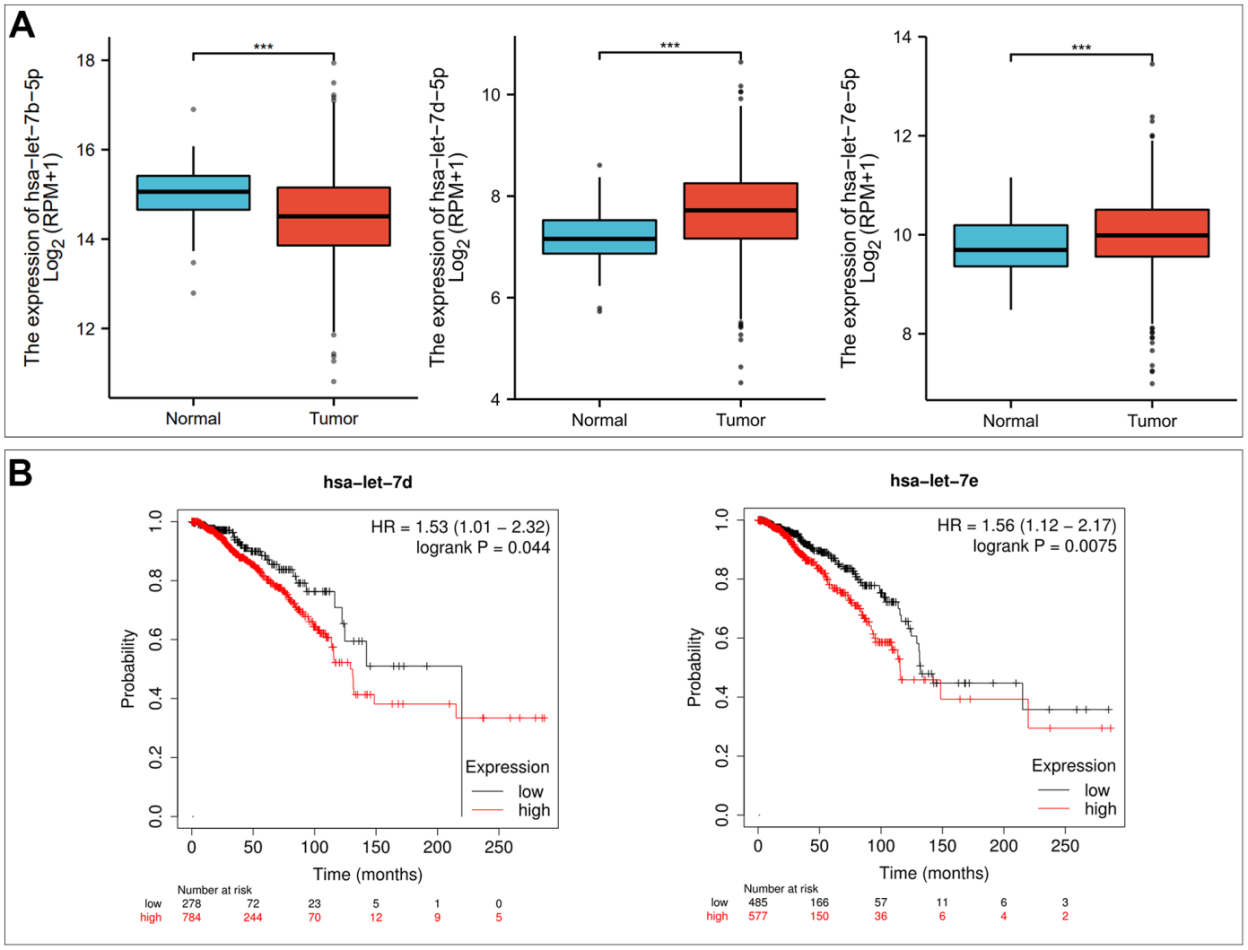
**Hsa-let-7d-5p and hsa-let-7e-5p are potential upstream binding miRNAs of P2RX1 in BC.** (**A**) Differential expression analysis of hsa-let-7b-5p, hsa-let-7d-5p and hsa-let-7e-5p in BC based on TCGA and GTEx database. (**B**) The prognostic value of hsa-let-7d-5p and hsa-let-7e-5p in BC evaluated by Kaplan-Meier plotter.

To explore the potential mechanism of the P2RX1 regulation, we firstly predicted possible upstream miRNAs and 11 upstream miRNAs that might be involved in the regulation of P2RX1 expression were discovered. Then we performed correlation analysis to determine the correlation between P2RX1 and these 11 miRNAs expression levels, and the results demonstrated that the expression of hsa-let-7c-5p (r = 0.223, p = 1.08E-13), hsa-let-7f-5p (r = 0.199, p = 3.88E-11), and hsa-let-7i-5p (r = 0.197, p = 5.96E-11) were positively correlated with the expression of P2RX1, while the expression of hsa-let-7b-5p (r = -0.169, p = 2.13E-08), hsa-let-7d-5p (r = -0.15, p = 7.07E-07), and hsa-let-7e-5p (r = -0.133, p = 1.03E-05) were inversely correlated with the expression of P2RX1 (Table 1). Previous studies have proved that miRNA usually negatively regulate the expression of targeted genes expression [16]. Therefore, the expression of hsa-let-7b-5p, hsa-let-7d-5p, and hsa-let-7e-5p in BC was assessed continuously. The results exposed that hsa-let-7b-5p was significantly downregulated in BC tissues, while hsa-let-7d-5p and hsa-let-7e-5p were significantly up-regulated which were positively correlated with the BC patients prognosis (p < 0.001) (Figure 4). Hsa-let-7d-5p and hsa-let-7e-5p were the most likely candidate miRNAs that might regulate P2RX1 expression.

**Table 1:** Correlation between predicted miRNA abundance and expression levels of P2RX1.

**miRNA**	**r**	**P value**
hsa-let-7a-5p	0.008	8.04E-01
**hsa-let-7b-5p**	**-0.169**	**2.13E-08**
hsa-let-7c-5p	0.223	1.08E-13
**hsa-let-7d-5p**	**-0.15**	**7.07E-07**
**hsa-let-7e-5p**	**-0.133**	**1.03E-05**
hsa-let-7f-5p	0.199	3.88E-11
hsa-miR-98-5p	-0.051	9.62E-02
hsa-let-7g-5p	-0.021	4.92E-01
hsa-let-7i-5p	0.197	5.96E-11
hsa-miR-4458	0.022	4.77E-01
hsa-miR-4500	0	1.00E+00

r, Spearman correlation coefficient.

### Prediction of upstream lncRNA of hsa-let-7d-5p and hsa-let-7e-5p

**Figure 5:**
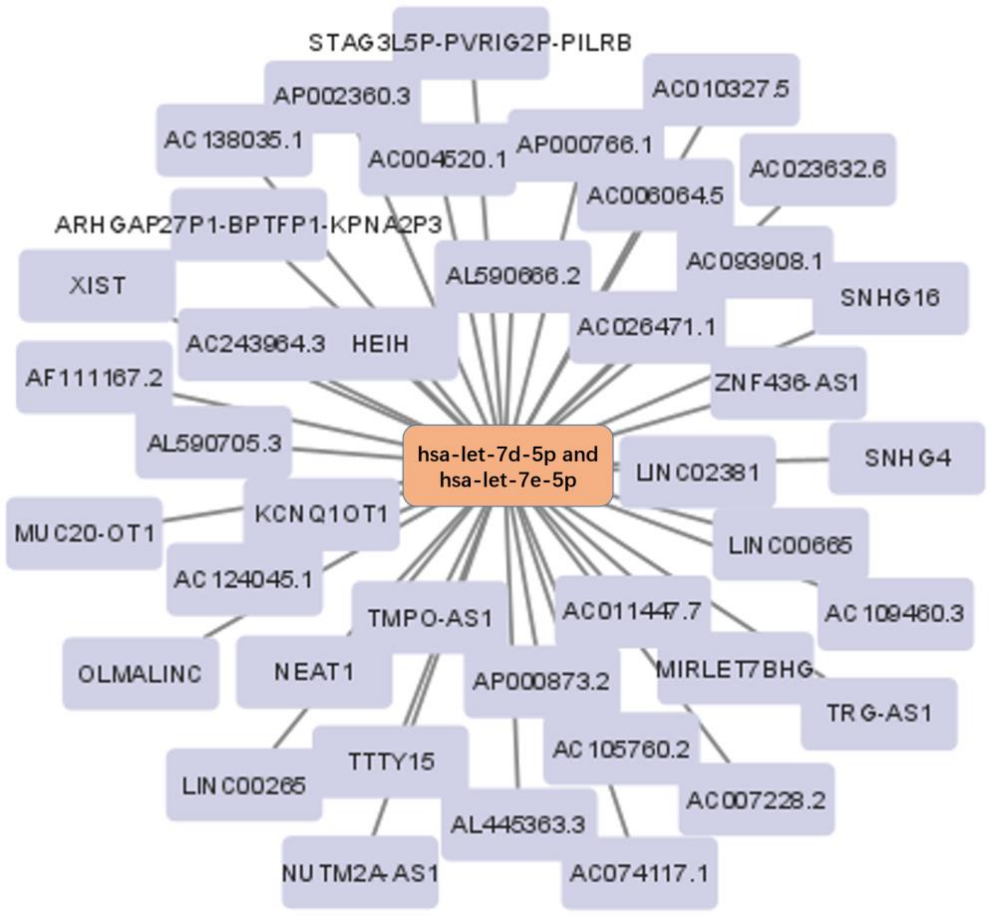
**The lncRNA-miRNA regulatory network.** The 40 lncRNAs predicted by the Starbase database were used to construct the lncRNA-miRNA regulatory network.

**Figure 6:**
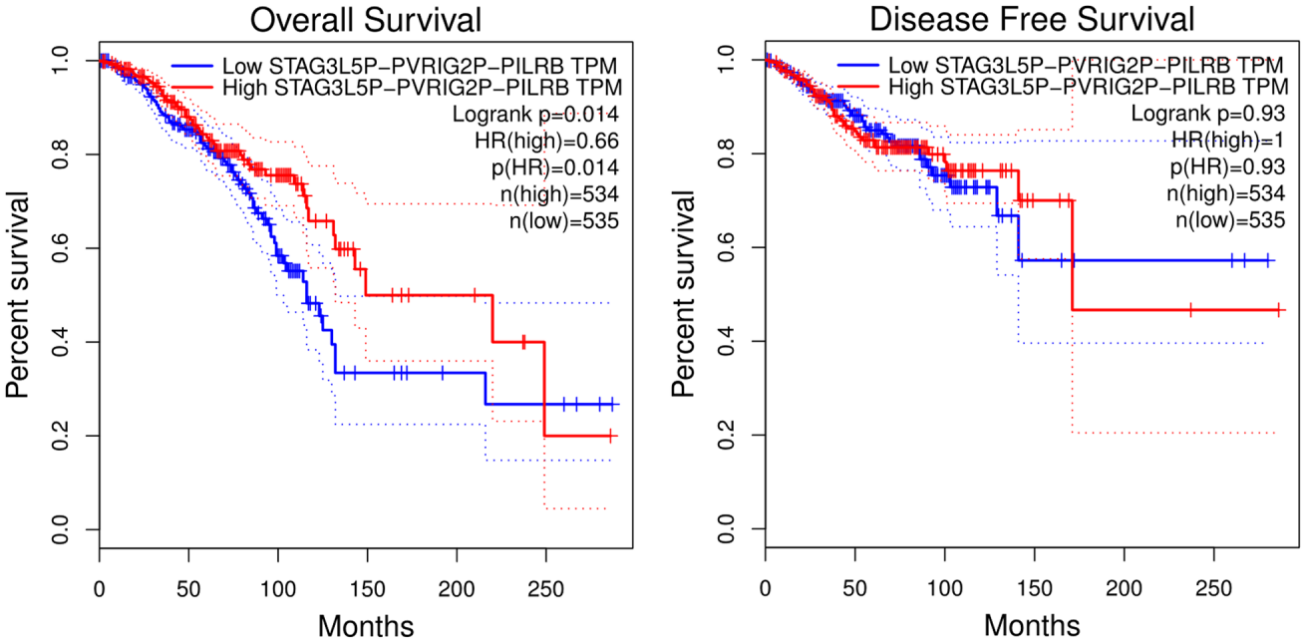
**Overall survival and disease-free survival analysis of STAG3L5P-PVRIG2P-PILRB expression in BC using the GIPIA platform.** High STAG3L5P-PVRIG2P-PILRB expression predicted better OS in BC patients (p = 0.014). But the two groups showed no statistical difference in DFS.

The Starbase database was used to predict the upstream lncRNA of hsa-let-7d-5p and hsa-let-7e-5p. Hsa-let-7d-5p and hsa-let-7e-5p belong to the same miRNA family with similar upstream regulatory lncRNAs. Among the 40 predicted lncRNAs (Figure 5), 19 lncRNAs were lowly expressed in BC tissues (Supplementary Figure 1). We further analyzed the prognostic values of these 19 lncRNAs in BC and revealed that low STAG3L5P-PVRIG2P-PILRB expression was associated with a significantly worse OS in BC patients (Figure 6). However, there was no statistical difference in DFS. STAG3L5P-PVRIG2P-PILRB might be the upstream lncRNA of hsa-let-7d-5p and hsa-let-7e-5p in BC.

### The construction and analysis of protein-protein interactions (PPI) network

**Figure 7:**
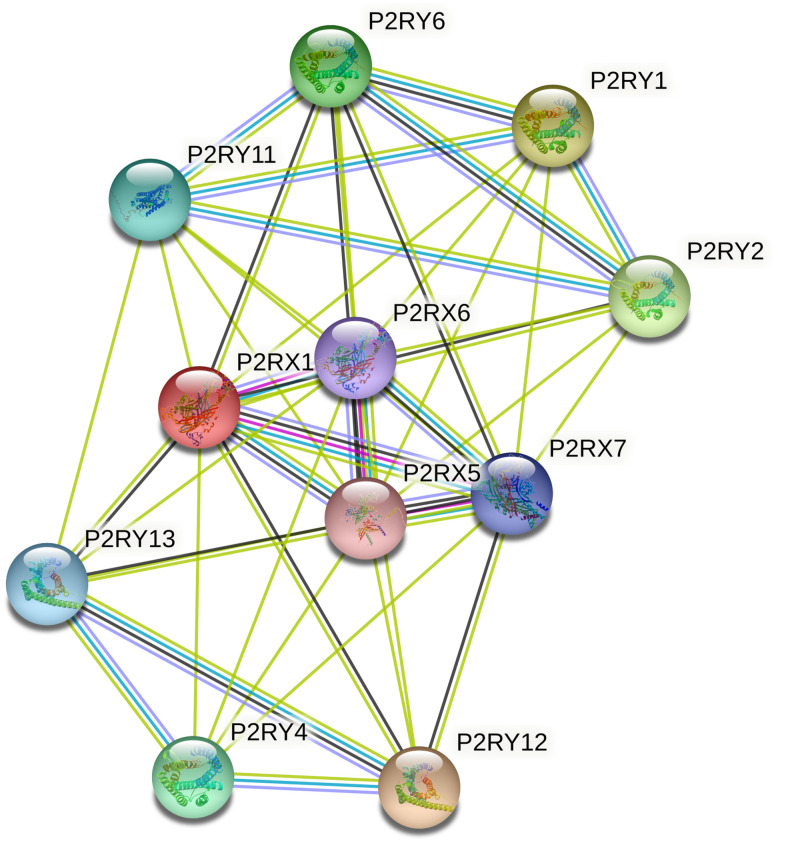
**The protein-protein interaction (PPI) network of P2RX1 based on the STRING database.** PPI network analysis showed the interaction of hub genes, including P2RX6, P2RX7, and P2RX5.

The PPI network complex constructed by the STRING database consisted of 11 nodes and 44 edges (Figure 7). Important nodes included P2RX6, P2RX7, and P2RX5. The average local clustering coefficient was 0.893. P2RX1 was involved in calcium-mediated signaling using extracellular calcium sources and G protein-coupled ADP and ATP receptor activity. KEGG analysis indicated that P2RX1 mainly contributed to neuroactive ligand-receptor interaction, Platelet activation, and Calcium signaling pathway.

### The correlation between P2RX1 and 6 types of immunocytes infiltration in BC

**Figure 8:**
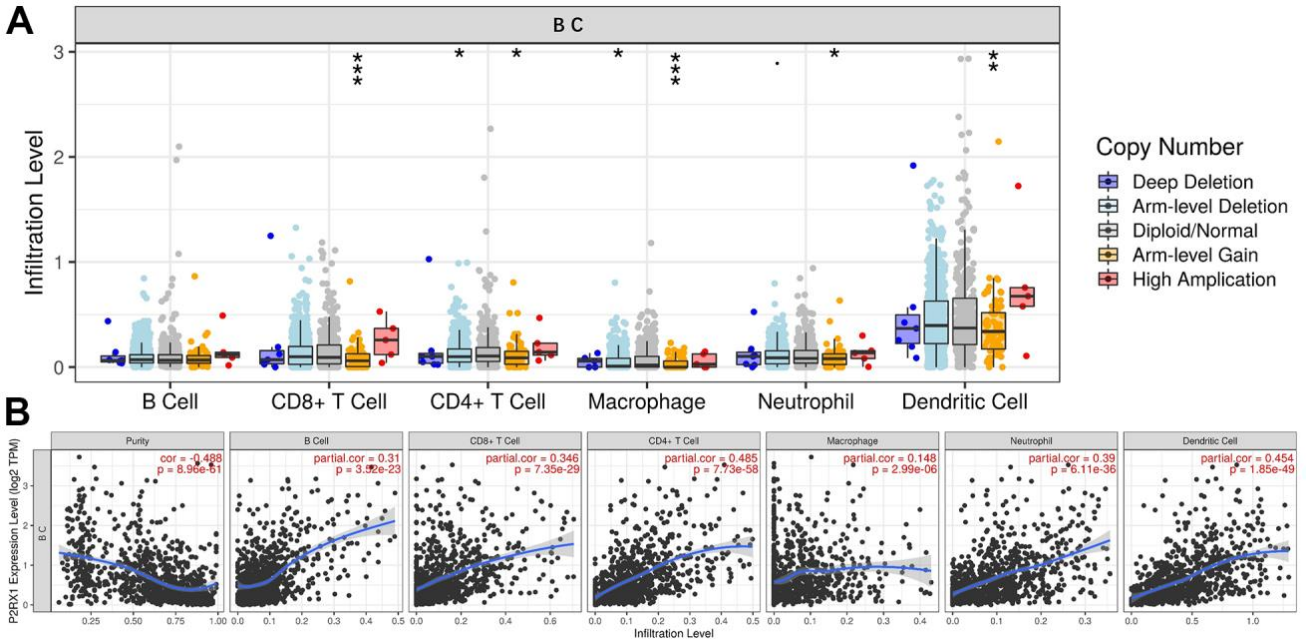
**The relationship between P2RX1 and immune cell infiltration.** (**A**) Infiltration levels of 6 immune cells with different copy number variations of P2RX1 in BC. (**B**) P2RX1 expression is positively correlated with 6 immune cells types evaluated by the TIMER database.

P2RX1 is highly expressed on the surface of immune cells, which may play an important role in immune cell infiltration. The significant differences in immunocytes infiltration levels under various copy numbers of P2RX1 were observed in Figure 8, which were mainly correlated with arm-level gain. Correlation analysis results indicated that the expression of P2RX1 positively correlates with 6 immune cells, including B cell, CD8+ T cell, CD4+ T cell, macrophage, neutrophil, and dendritic cell in BC. Further investigation of immune biomarkers shown in Supplementary Figure 2 validated that P2RX1 expression was significantly positively correlated with B cell’ biomarkers (CD27, CD19), CD8+ T cell’ biomarkers (CD8A and CD8B), CD4+ T cell’ biomarker (CD4), M1 macrophage’ biomarkers (CD68, CXCL10), M2 macrophage’ biomarkers (CCL18, CD163), neutrophil’ biomarkers (ITGAM), and dendritic cell’ biomarkers (CD83, CD1c). These findings support the positive correlation between P2RX1 and immunocytes infiltration in BC.

### The correlation between P2RX1 and immune checkpoints in BC

**Figure 9:**
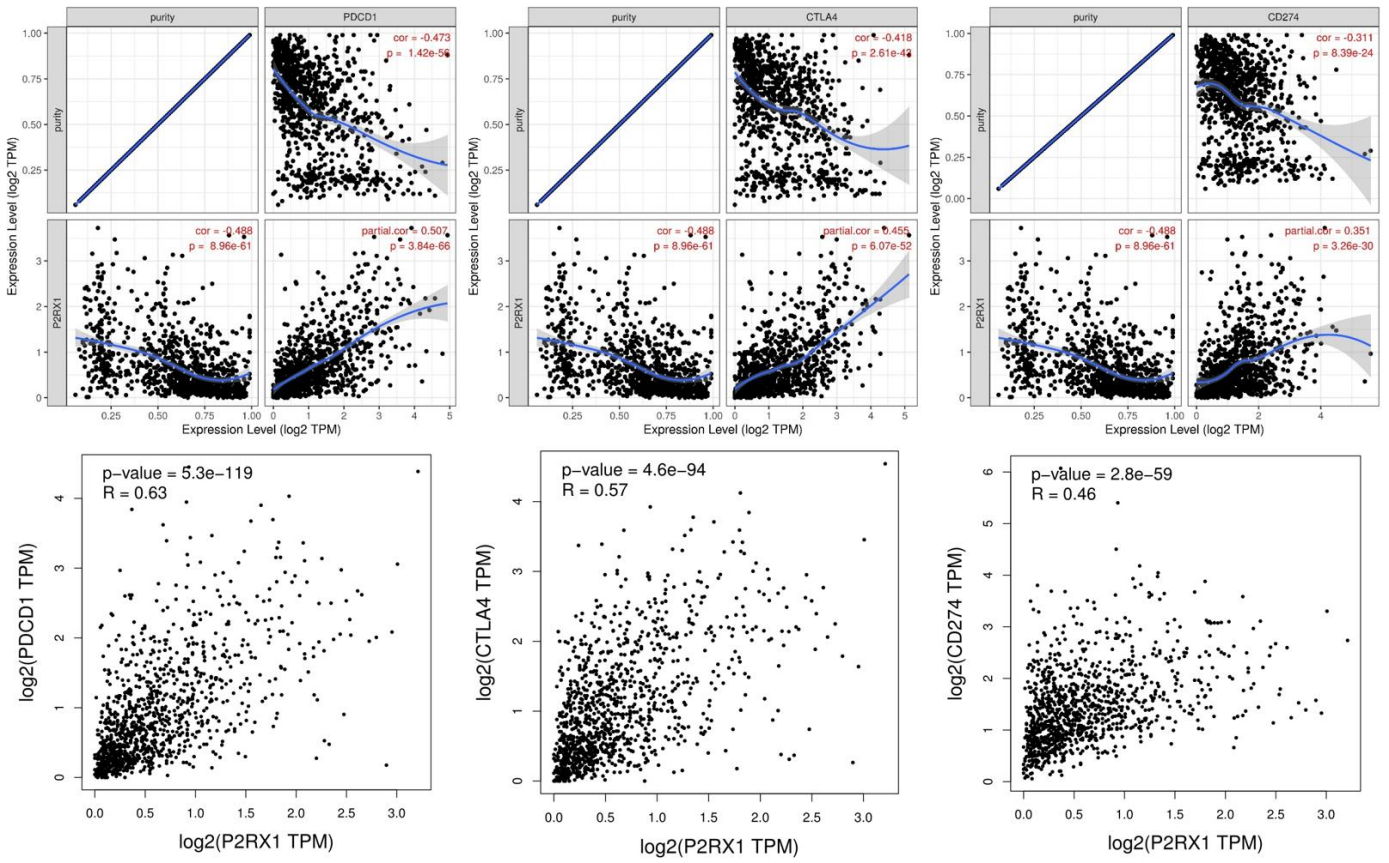
The expression of P2RX1 is significantly positively correlated with PDCD1, CTLA4, and CD274 in BC evaluated by TIMER and GIPIA database.

At present, the main immune checkpoints are PD-1 (PDCD1), PD-L1 (CD274), and CTLA4 which are important biomarkers for predicting the efficacy of immunotherapy. As shown in Figure 9, P2RX1 expression was significantly positively correlated with PDCD1 (r = 0.507, p = 3.84e-66), CTLA4 (r = 0.455, p = 6.07e-52) and CD274 (r = 0.351, p = 3.26e-30) in BC after adjusted by tumor purity. Data from GIPIA showed results similar to those from the TIMER database which suggested P2RX1 may participate in the process of tumor immunomodulation.

## DISCUSSION

P2RX1 belongs to a family of P2X purinergic receptors activated by extracellular ATP, which is highly expressed at the surface of various cells [9, 14]. Previous studies have shown that P2RX1 is associated with the carcinogenesis, and prognosis of pancreatic cancer and lung cancer [14, 17]. Given the important role of P2RX1 in immune regulation, this protein has received increasing attention in recent years. But few studies have been investigated in the field of BC.

First, expression data for P2RX1 in pan-cancer were accessed from TCGA and GTEx databases and identified the differential expression of P2RX1 in 22 pairs of human tumors and the corresponding adjacent para-cancerous tissues. Among them, P2RX1 expression was significantly lower in 12 tumor tissues types compared with the para-cancerous tissues. The survival analysis of these 12 human tumors showed that a higher P2RX1 expression predicts better OS and DFS in BC patients. Therefore, P2RX1 can be used as a biomarker for the diagnosis and prognosis of BC. In many other tumor types, P2RX1 has also been shown to have prognostic value, such as lung cancer [15] and pancreatic cancer [14], which is consistent with the findings in this study.

Regulation of P2RX1 expression by ncRNA may be completed through the ceRNA-related regulatory mechanisms [18]. Informed through target gene prediction programs, hsa-let-7d-5p and hsa-let-7e-5p were the most potential upstream regulatory miRNAs of P2RX1. Hsa-let-7d-5p has been found to be highly expressed in BC, especially metastatic BC [19–21]. Danielle et al. suggested that hsa-let-7d-5p could be a potential biomarker for hereditary breast tumors [22]. Moreover, hsa-let-7d-5p has also been considered to be associated with the sensitivity of BC drugs [23]. Similarly, high expression of hsa-let-7e-5p previously showed an unfavorable prognosis in BC [18, 24].

The potential lncRNA of hsa-let-7d-5p and hsa-let-7e-5p/P2RX1 axis was STAG3L5P-PVRIG2P-PILRB, which has been reported to be up-regulated in renal papillary cell carcinoma and may be associated with poor prognosis [25]. Tait et al. found that the dysfunction of STAG3L5P-PVRIG2P-PILRB may correlate with colorectal cancer carcinogenesis [26]. The STAG3L5P-PVRIG2P-PILRB/hsa-let-7d-5p and hsa-let-7e-5p/P2RX1 axis are the underlying regulatory pathways of BC, which affect the carcinogenesis and prognosis of BC.

In addition, this study analyzed the PPI related to P2RX1 using the STRING database and found that P2RX1 was correlated with P2RX6, P2RX7, and P2RX5 which all belong to the P2X purinergic receptor family. Wang et al. reported that P2RX7, which is down-regulated in lung cancer, is a good prognostic factor [17]. But other studies suggested that P2RX7 is a poor prognosis factor increasing the aggressiveness and metastasis [27, 28]. These results indicate that purinergic receptors exhibit complex functions in cancer initiation and development. Notably, purinergic receptors may influence tumor prognosis by regulating immune cell function.

Although P2RX1 is highly expressed in para-cancerous tissues, this study found that normal human mammary epithelial cells express lower P2RX1, compared to human breast cancer cells. Therefore, the high expression of P2RX1 in para-cancerous tissues may be attributed to immune cells. It has been documented that the low P2RX1 expression of immune cells contributes to a condition of tumor immunosuppression, thereby promoting liver metastasis of pancreatic cancer [14]. P2RX1 activation promotes the activation of CD4+ T cell [29] and neutrophils [10], which may be related to the less glycolysis but more oxidation of P2RX1 knockout neutrophils [14]. In BC, P2RX1 is activated by ATP that causes transmembrane transport of ions, thereby activating various immune cells. This study indicated that P2RX1 is significantly positively correlated with tumor-infiltrating immune cells and their biomarkers in BC, including B cell, CD8+ T cell, CD4+ T cell, macrophage, neutrophil, and dendritic cell, revealing the important role of P2RX1 in BC immune modulation.

We further analyzed the correlation between P2RX1 and immune checkpoints, which are essential biomarkers for the efficacy outcomes of immunotherapy in BC [2]. The results showed that P2RX1 was significantly positively correlated with PDCD1, CTLA4, and CD274 in BC, suggesting that P2RX1 has the potential to become a new immune checkpoint.

In summary, this study revealed that compared with para-cancerous tissues, P2RX1 expression in a variety of tumors, including BC, was significantly decreased, and the P2RX1 expression level was strongly associated with clinical outcomes. We analyzed the upstream regulators and the PPI of P2RX1 in BC and revealed that P2RX1 was significantly positively correlated with immune cell infiltration and immune checkpoints. These results show that P2RX1 may be a promising biomarker and therapeutic target for BC, and more experimental and clinical studies are warranted to validate these findings, which may facilitate the development of novel therapies for BC.

## MATERIALS AND METHODS

### Data acquisition and pan-cancer analysis

We downloaded transcriptome and clinical data of pan-cancer from the TCGA database (https://tcga-data.nci.nih.gov/tcga/) and GTEx database (http://www.gtexportal.org/). After sample normalization by Toil [30], differential expression analysis was performed on pan-cancers. Patients were classified into P2RX1 high and low expression groups based on the 50th percentile.

### Differential expression and prognosis analysis

GEPIA is a website based on TCGA and GTEx data, using the output of a standard processing pipeline for high-throughput RNA sequencing data (http://gepia.cancer-pku.cn/) [31]. We used GEPIA to evaluate the expression difference of P2RX1 between tumor (TCGA samples) and normal (GTEx samples) tissues in 33 tumor tissues, as well as prognostic profiles including OS and DFS. GEPIA was also used to analyze the prognostic value of the candidate lncRNAs in BC patients. In addition, the correlation between P2RX1 and immune checkpoint inhibitors (PDCD1, CD274, and CTLA4) was determined using GEPIA.

### Real-time quantitative reverse transcription PCR

Normal human mammary epithelial cells (MCF 10A) and human breast cancer cells (MDA-MB-231 and MCF-7) were bought from Procell Life Science and Technology Co., Ltd (Wuhan, China) and were authenticated with STR assays and tested negative for mycoplasma infection. Total RNA was extracted from MCF 10A, MDA-MB-231, and MCF-7 cells with an RNA extraction kit (Baidai, China), and RNA concentration was determined with Nanodrop (Thermo Fisher, USA). Reverse transcription was performed using ReverTra Ace qPCR RT Master Mix (TOYOBO, Japan). RT-qPCR was performed on a QuantStudio 6 Pro Real-Time PCR Systems (Thermo Fisher, USA) using the SYBR Green PCR kit (TOYOBO, Japan). The normalized internal reference was GAPDH mRNA. The relative expression of P2RX1 was calculated using the 2-ΔΔCT method. The primers sequence was as follows: forward, ATGGTGCTGGTGCGTAATAAG, and reverse, GGAAGACGTAGTCAGCCACA.

### Western blot analysis

Normal human mammary epithelial cell line MCF 10A was cultured in DMEM containing 5% horse serum, 20ng/ml Epidermal Growth Factor, 0.5μg/ml Hydrocortisone, 10μg/ml Insulin, and 1% Non-Essential Amino Acids (CM-0525, Procell, China). The human breast cancer cell line MDA-MB-231 was cultured in Leibovitz's L-15 medium (PM151010, Procell, China) containing 10% fetal bovine serum (Gibco, USA). The human breast cancer cell line MCF-7 was cultured in DMEM medium (Gibco, USA) containing 10% fetal bovine serum (Gibco, USA). MCF 10A and MCF-7 were maintained in a humidified incubator at 37° C and 5% CO2, and MDA-MB-231 was maintained in a humidified incubator at 37° C.

Proteins from normal human mammary epithelial cells and human breast cancer cells were collected using RIPA buffer (Beyotime, China) and quantified using the BCA protein assay kit (Beyotime, China). Proteins were separated using 10% sodium dodecyl sulfate-polyacrylamide gel electrophoresis and transferred to polyvinylidene fluoride membranes. Membranes were blocked with 5% nonfat dry milk and washed with Tris-buffered saline containing 0.1% Tween 20.

The following primary antibody was used for overnight incubation at 4° C: rabbit anti-P2RX1 (1:2000, DF9728, Affinity, China) and mouse anti-GAPDH (1:4000, T0004, Affinity, China). Secondary antibodies were goat anti-rabbit IgG (H+L) HRP conjugate and goat anti-mouse IgG (H+L) HRP (horseradish peroxidase) conjugate (anti-rabbit, 1:4000, Affinity, China, and anti-mouse, 1:20000, Beijing Zhongshan). ECL hypersensitive luminescent liquid (Millipore, USA) and Multiplex Fluorescence Western blot imaging system (ProteinSimple, USA) were used to detect protein strips.

### Associated miRNA prediction

In this study, we used 5 target gene prediction programs to predict potential upstream binding miRNAs of P2RX1, namely miRWalk, TargetScan, miRDB, RNA22, and miRmap. The miRNAs predicted by these five programs at the same time were considered candidate miRNAs and were used for further analysis.

### Starbase database analysis

Starbase (http://starbase.sysu.edu.cn/), an online database for ncRNA functions and their coordinated regulatory networks, was used to predict candidate lncRNA, and analyze the correlation between miRNA and P2RX1, as well as candidate lncRNA and P2RX1 [32].

### Kaplan-Meier plotter database analysis

Kaplan-Meier plotter database (http://kmplot.com/analysis/) was used to analyze the effect of potential miRNAs on the survival of BC patients who were divided into two groups based on the median expression of miRNAs. Kaplan-Meier survival plots were constructed and the log-rank p value was calculated.

### STRING database analysis

The STRING tool (https://string-db.org) is a database of known and predicted PPI. STRING database was used to investigate the PPI network of P2RX1 and analyze the functional interactions. Nodes and edges of different colors indicate the relationship between proteins and proteins.

### TIMER database analysis

TIMER database (https://cistrome.shinyapps.io/timer/) is a tool for the analysis of immune infiltrates. TIMER database was used to analyze the relationship between P2RX1 expression and tumor immune infiltrating cells including B cells, CD4+ T cells, CD8+ T cells, neutrophils, macrophages, and dendritic cells. Then the immune checkpoints were also evaluated to explore their correlation with P2RX1 expression.

### Statistical analysis

The expression difference of P2RX1 in pan-cancer was compared by the Wilcoxon rank sum test. The survival differences were analyzed by log-rank test and Kaplan-Meier method. The significant difference was determined at p < 0.05. These analyses were conducted in R Version 3.6.1 and Stata/MP Version 13.1.

## Supplementary Material

Supplementary Figures
