# Supplementary material for: NcRNAs-mediated P2RX1 expression correlates with clinical outcomes and immune infiltration in patients with breast invasive carcinoma
Source: Aging (Albany NY). 2022 May 18;14(10):4471–85. doi: 10.18632/aging.204087 (PMC9186779; doi:10.18632/aging.204087)
Supplement: Supplementary Figures [file aging-14-204087-s001.pdf]

SUPPLEMENTARY FIGURES

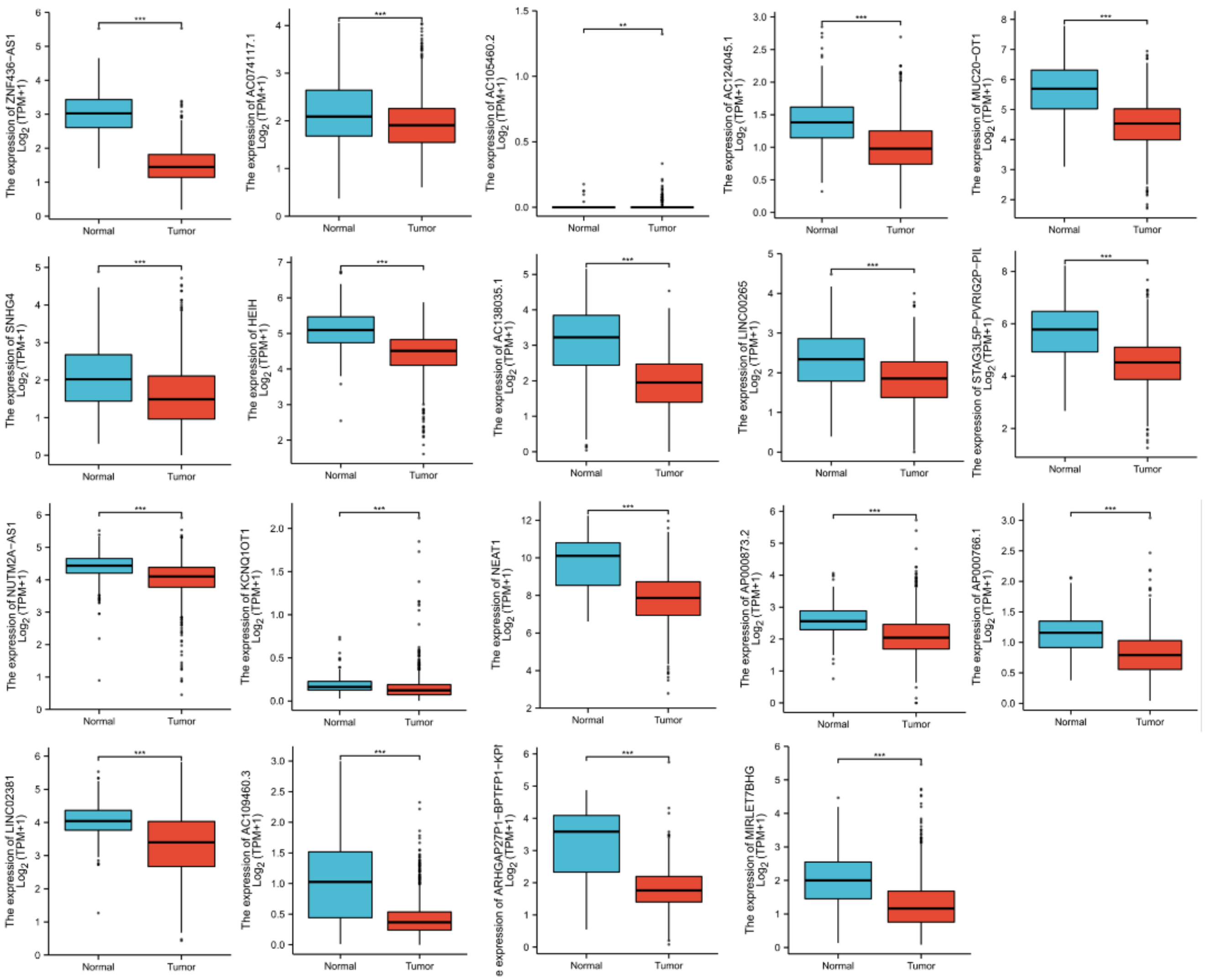

Supplementary Figure 1. Low expression of 19 lncRNAs in breast invasive carcinoma tissues than para-cancerous tissues, including ZNF436-AS1, AC074117.1, AC105760.2, AC124045.1, MUC20-OT1, SNHG4, HEIH, AC138035.1, LINC00265, STAG3L5P-PVRIG2P-PILRB, NUTM2A-AS1, KCNQ1OT1, NEAT1, AP000873.2, AP000766.1, LINC02381, AC109460.3, ARHGAP27P1-BPTFP1-KPNA2P3, and MIRLET7BHG.

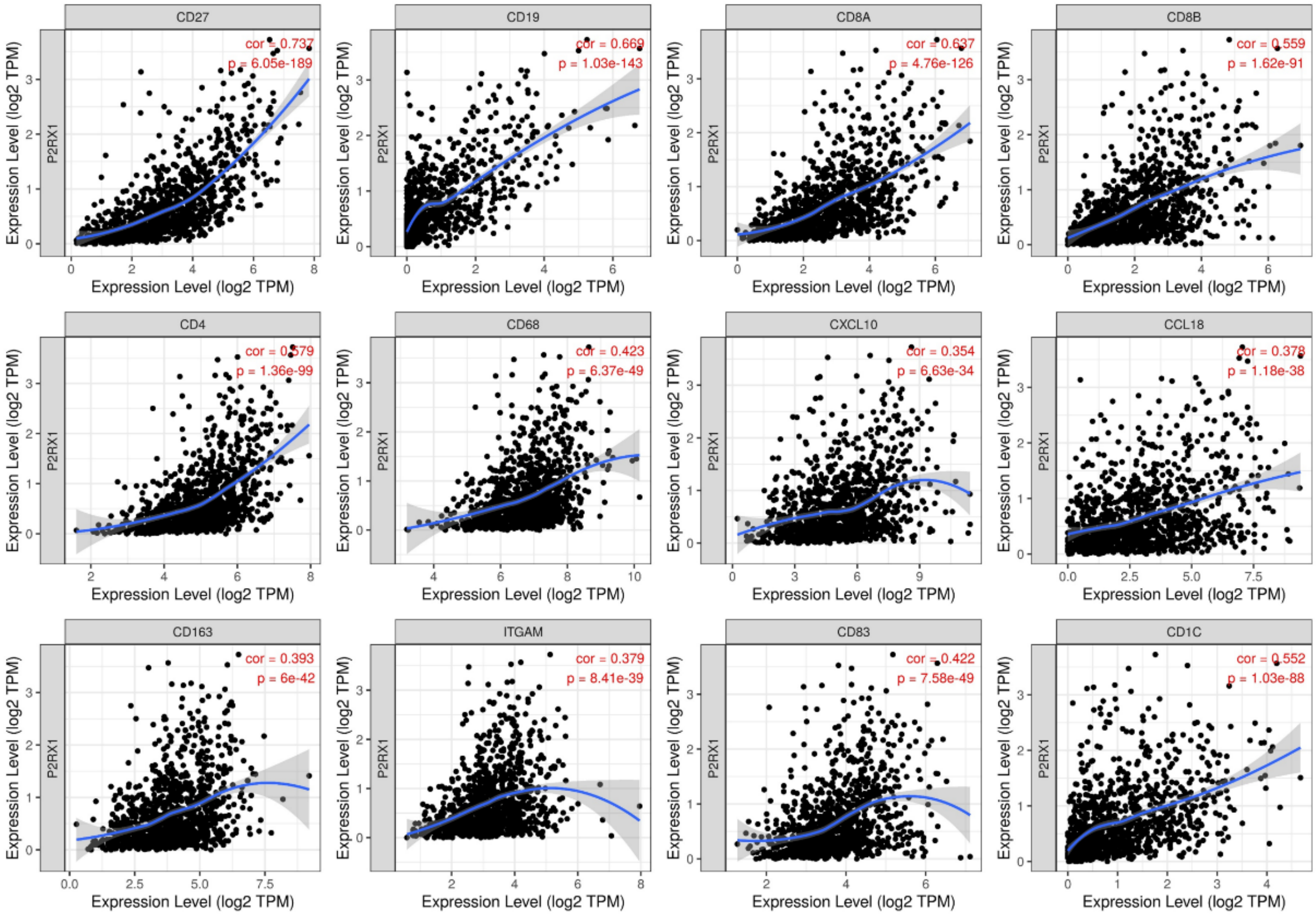

Supplementary Figure 2. The relationship between P2RX1 expression and immune biomarkers in breast invasive carcinoma. The expression of P2RX1 was significantly positively correlated with B cell' biomarkers (CD27, CD19), CD8+ T cell' biomarkers (CD8A and CD8B), CD4+ T cell' biomarker (CD4), M1 macrophage' biomarkers (CD68, CXCL10), M2 macrophage' biomarkers (CCL18, CD163), neutrophil' biomarkers (ITGAM), and dendritic cell' biomarkers (CD83, CD1c).
